# Constraint and diversification of developmental trajectories in cichlid facial morphologies

**DOI:** 10.1186/s13227-015-0020-8

**Published:** 2015-06-28

**Authors:** Kara E. Powder, Kayla Milch, Garrett Asselin, R. Craig Albertson

**Affiliations:** Department of Biology, 221 Morrill Science Center South, University of Massachusetts, 611 North Pleasant Street, Amherst, MA 01003 USA

**Keywords:** Developmental trajectory, Geometric morphometrics, Phenotypic variation, Craniofacial, Cichlid, Ontogeny

## Abstract

**Background:**

A major goal of evolutionary biology is to understand the origins of phenotypic diversity. Changes in development, for instance heterochrony, can be a potent source of phenotypic variation. On the other hand, development can also constrain the spectrum of phenotypes that can be produced. In order to understand these dual roles of development in evolution, we examined the developmental trajectory of a trait central to the extensive adaptive radiation of East African cichlid fishes: craniofacial adaptations that allow optimal exploitation of ecological niches. Specifically, we use geometric morphometric analysis to compare morphological ontogenies among six species of Lake Malawi cichlids (*n* > 500 individuals) that span a major ecomorphological axis. We further evaluate how modulation of Wnt signaling impacts the long-term developmental trajectory of facial development.

**Results:**

We find that, despite drastic differences in adult craniofacial morphologies, there are general similarities in the path of craniofacial ontogeny among species, suggesting that natural selection is working within a conserved developmental program. However, we also detect species-specific differences in the timing, direction, and/or duration of particular developmental trajectories, including evidence of heterochrony. Previous work in cichlids and other systems suggests that species-specific differences in adult morphology are due to changes in molecular signaling pathways that regulate early craniofacial development. In support of this, we demonstrate that modulation of Wnt signaling at early stages can shift a developmental trajectory into morphospace normally occupied by another species. However, without sustained modulation, craniofacial shape can recover by juvenile stages. This underscores the idea that craniofacial development is robust and that adult head shapes are the product of many molecular changes acting over extended periods of development.

**Conclusions:**

Our results are consistent with the hypothesis that development acts to both constrain and promote morphological diversity. They also illustrate the modular nature of the craniofacial skeleton and hence the ability of selection to act upon distinct anatomical features in an independent manner. We propose that trophic diversity among cichlids has been achieved via shifts in both specific (e.g., stage-specific changes in gene expression) and global (e.g., heterochrony) ontogenetic processes acting within a conserved developmental program.

**Electronic supplementary material:**

The online version of this article (doi:10.1186/s13227-015-0020-8) contains supplementary material, which is available to authorized users.

## Background

Development plays dual roles with respect to determining evolutionary potential. On the one hand, natural selection acts on phenotypic variation that is produced by heritable alterations in development. Alternatively, the process of development may act to constrain the types of phenotypes that can evolve, specifically the direction and the amount of variation that can be generated. Understanding how development both produces and constrains phenotypic variation is central to understanding morphological evolution, as well as evolvability, the capacity of an organism to evolve in the future [1–5]. For instance, changes in developmental trajectories, including heterochrony [6–8], have been implicated in evolutionary transitions between Neanderthals and modern humans [9], dinosaurs and birds [10], and avian and non-avian amniotes [11] and in producing adaptive variation in limb length among lizards [12].

The development of complex morphological traits involves the integration of multiple molecular and cellular pathways, different tissues, distinct functional and anatomical units, and environmental interactions over an extended period of time [13, 14]. For instance, during facial development, cranial neural crest cells, the cellular origins of most of the facial skeleton in vertebrates [15–21], must coordinate complex molecular and morphogenetic patterns to properly form, migrate, proliferate, and differentiate into cartilage and bone. This involves multiple gene products and developmental signaling pathways (reviewed in [22–24]), requires tissue-tissue interactions (e.g., of neural and non-neural ectoderm during induction [24] and ectoderm and mesenchyme during facial outgrowth [25]), and is influenced by the development of other anatomically distinct units (e.g., growth of the brain [26, 27]). Alterations within any of these developmental mechanisms can produce phenotypic variation. For example, activity of the Wnt signaling pathway is critical for multiple stages of facial [22] and bone development [28]. Variation in the expression patterns and levels of this pathway during early facial patterning and chondrogenesis have been associated with microevolution of craniofacial structures among birds [29] and fish [30], as well as macroevolution of facial structures between birds and mammals [31]. Finally, the craniofacial skeleton can be remodeled over time to accommodate shifts in foraging niches, and thus, its geometry is strongly influenced by the environment (e.g., hardness of diet and feeding mechanics [30, 32, 33]).

East African cichlids exhibit one of the most impressive adaptive radiations, with hundreds of species radiating within the last million years [34]. Pivotal to this radiation are species-specific craniofacial features that facilitate ecological specialization [35], making cichlids an ideal model to address the role of development in the production of phenotypic variation. The primary axis of craniofacial variation in cichlids, like many fish lineages, distinguishes two primary feeding mechanisms [36]. Species on one end of this axis are pelagic feeders that forage on mobile prey, often from the water column via suction feeding and are characterized by a long mandible, a shallow craniofacial profile, and isognathus jaws. Species on the opposite end of this spectrum are benthic feeders that forage by biting, crushing, scraping, and/or picking prey from rocks and are characterized by a short mandible, a rounded/steep craniofacial profile, and ventrally directed jaws [30, 36]. Note, our use of either “pelagic” or “benthic” to describe species within this study is meant to describe foraging mode (i.e., sucking or biting, respectively), not habitat preference.

**Fig. 1:**
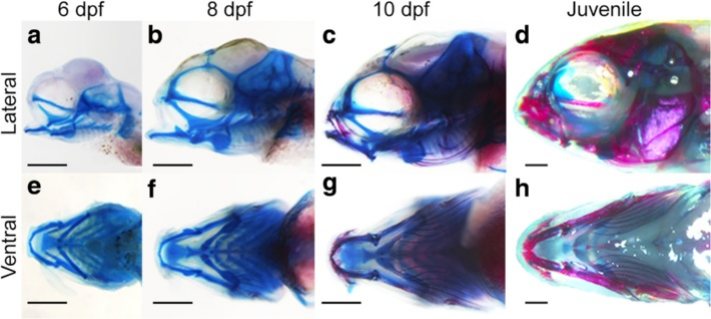
Time course of craniofacial development analyzed. **a**–**d L**ateral and **e**–**h** ventral views of time points analyzed. Scale = 500 μm

**Fig. 2:**
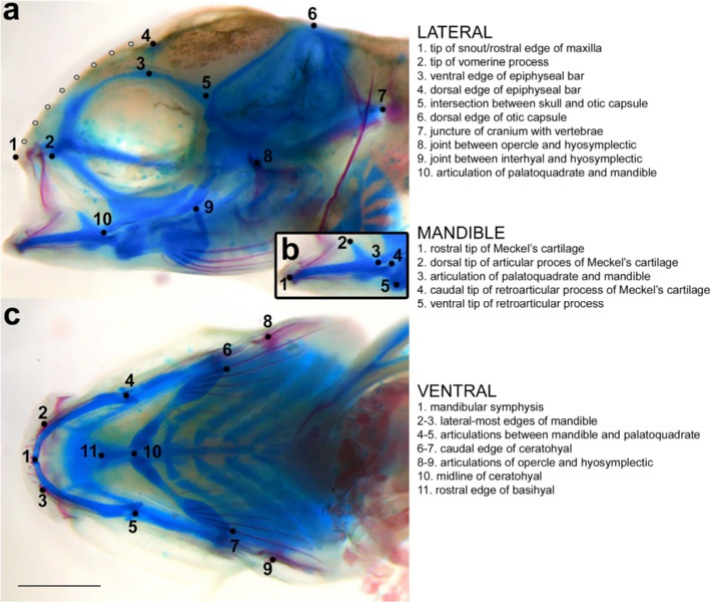
Landmarks and semilandmarks used in morphometric analysis. Representative larvae are shown with the location and description of landmarks used to analyze **a** lateral, **b** mandible, and **c** ventral development. For lateral development, eight semilandmarks (*gray*) were evenly placed between landmarks 1 and 4 to capture the slope of the craniofacial complex

Here, we assess craniofacial ontogenies for six species of Lake Malawi cichlids that span the pelagic-benthic ecomorphological axis [36]: *Aulonocara* sp. (Au), *Tramitichromis* sp. (Tra), *Maylandia zebra* (MZ), *Tropheops tropheops* (TT), *Tropheops* sp. “red cheek” (TRC), and *Labeotropheus fuelleborni* (LF), listed from the most pelagic to the most benthic species (Table 1). The period examined spans a wide window of craniofacial development, from the establishment of craniofacial structures as cartilaginous precursors through when fish begin to forage, and includes larval and juvenile stages (Fig. 1). We used geometric morphometrics to analyze shape variation for three functionally relevant aspects of the craniofacial skeleton: (1) the mandible, (2) the ventral view of the pharyngeal skeleton including the mandible and branchial cartilages/bones, and (3) the lateral view of most of the craniofacial complex including the upper jaw, orbit, and the brain case (Fig. 2).

**Table 1:** Species examined span the benthic-pelagic ecomorphological axis. List of species and treatments used in analyses, including habitat and feeding strategy based on [47]

	Species (and treatment, as applicable)	Habitat	Feeding strategy	Color in figures
	*Aulonocara* sp. (Au)	Sand	Sonar hunting	
	*Tramitichromis* sp. (Tra)	Sand	Sifting	
	*Maylandia zebra* (MZ)	Rock	Suction/combing	
	*Tropheops tropheops* (TT)	Rock	Biting: nip and twist	
	*Tropheops* sp. “red cheek” (TRC)	Rock	Biting: nip and twist	
	*Labeotropheus fuelleborni* (LF)	Rock	Biting: scraping	
	*Maylandia zebra* 8 mM LiCl, 6 hr at 5 dpf			
	*Tropheops tropheops* 8 mM LiCl, 6 hr @ 5 dpf			
	*Tropheops tropheops* 8 mM LiCl, 6 hr @ 5,10,16 dpf			
	*Tropheops tropheops* 250 uM LiCl, continuously starting @ 5 dpf

We predicted that species-specific craniofacial shapes may be generated by a combination of distinct developmental mechanisms. For example, differences in morphology may arise due to variation in early patterning events such as neural crest cell development and/or chondrogenesis (e.g., [37]). These early differences would then be elaborated into distinct adult forms via parallel developmental trajectories. Alternatively, craniofacial shapes may be largely conserved early and diverge through ontogeny via species-specific trajectories. Finally, divergence in both early patterning events and long-term ontogenetic trajectories may underlie variation in cichlid craniofacial shape. A rigorous assessment of facial shape through ontogeny will provide greater insights into how and when development produces morphological variation on which natural selection can act, as well as the dynamics of craniofacial development.

## Methods

### Animals

All rock-dwelling cichlids were maintained and used according to guidelines and protocols approved by the Institutional Animal Care and Use Committee, including Ethical Committee, at the University of Massachusetts Amherst. LF, MZ (also called *Metriaclima zebra*), TT, and TRC were maintained and bred at 28.5 °C in a 14-h light/10-h dark cycle. Cichlid species were collected from Lake Malawi and reared in 40-gal glass aquaria. Larvae (*F*_1_- to *F*_5_-derived from wild-caught stocks) were obtained by natural matings and, following collection from mouth-brooding females, were incubated in 1-L flasks with system water plus 2–3 drops of methylene blue at 28.5 °C. The larvae were staged based on caudal fin anatomy according to [38] and collected at 6 dpf (days post fertilization, stage 18), 8 dpf (stage 22), and 10 dpf (stage 24). The fish were moved to 10-gal glass aquaria once yolk was absorbed (approximately 15 dpf) and raised for approximately 1 month, until juveniles were a standard length (SL, length from tip of the snout to the base of the caudal fin rays) of 1.2–1.6 cm (mean = 1.4 cm). These time points were chosen because the larval stages (6–10 dpf) represent a critical period of facial development, including when the facial bones are initially established. By juvenile stages (SL of 1.2–1.6 cm), a large portion of bone development is complete and fish are eating independently. Sand-dwelling cichlid (*Aulonocara* sp. and *Tramitichromis* sp.) samples were raised in a similar manner, as described in [39]. Number of specimens and families used are listed in Additional file 1: Table S1; specimens were evenly sampled from all families. Note that we did not have samples from *Tramitichromis* sp. at 6 dpf.

### Fixation and skeletal staining

The specimens were sacrificed by overdose with tricaine methanesulfonate (MS-222, Aquatic Ecosystems Inc.), fixed overnight at room temperature in 4 % paraformaldehyde (Sigma) in 1× phosphate-buffered saline (PBS), and dehydrated to 70 % ethanol. Skeletal elements were stained using Alcian Blue and Alizarin Red for cartilage and bone, respectively, based on [40]. The larvae were incubated at room temperature overnight in a solution of 150 uL 0.5 % Alizarin Red S (Sigma) in water plus 5 mL 0.02 % Alcian Blue 8GX (Sigma) and 60 mM MgCl_2_ in 70 % ethanol. The samples were bleached using 3 % hydrogen peroxide and 2 % potassium hydroxide (KOH) until melanocytes turned from black to brown; any bubbles formed during this process were removed manually. The tissues were cleared by transitioning through the following series: 25 % glycerol and 0.25 % KOH (>30 min), 50 % glycerol and 0.25 % KOH (>2 h), and 80 % glycerol (>2 h and storage). The eyes were manually removed to allow visualization of internal structures. The samples were positioned in 80 % glycerol and photographed with a scale bar using a Leica DFC450 camera mounted to a Leica MF15 stereomicroscope in both lateral and ventral views. Three different aspects of the craniofacial skeleton were analyzed for shape variation: the ventral view (Fig. 2c), including the mandible and ceratobranchial cartilages/bones; the lateral view (Fig. 2a), incorporating the upper jaw through the neurocranial region; and the mandible (Fig. 2b), which was extracted from the lateral image but analyzed independently to eliminate variation introduced by degree of jaw opening at fixation.

### Geometric morphometrics

The position of homologous anatomical landmarks (LMs) (Fig. 2) was collected from photos using the software tpsDig2 [41]. For the lateral view, semilandmarks were also collected as a curve that defined the slope of the craniofacial profile (Fig. 2a). These data were reduced to eight evenly spaced landmarks and subsequently defined as semilandmarks using the software tpsUtil [41]. The program tpsRelw [41] was used to conduct Procrustes superimposition of landmarks and semilandmarks using a chord-distance (Procrustes distance)-based “sliders” method, which removed variation due to size, rotation, and position, leaving only variation due to shape. TpsRelw was also used to generate partial warps from landmark data and perform a principal component analysis (PCA) on these variables. The samples were analyzed both through ontogeny (including samples at 6 dpf, 8 dpf, 10 dpf, and juveniles) and within a single developmental time point (i.e., among individuals of the same age). For mandible ontogeny, the 10-dpf time point was omitted; at this point, the cartilage of the coronoid process is being resorbed as the bone is formed, resulting in unreliable placement of landmarks.

### Comparison of developmental trajectories and statistics

The shape, length, and orientation of developmental trajectories were quantified and statistically compared using the trajectory analysis function in the geomorph package for R [42, 43]. Pairwise differences were assessed using 10,000 residual randomization permutations, with and without Bonferroni correction. We also conducted multivariate analysis of variance (MANOVA) tests on partial warp scores (principal components [PC] 1–3 combined) to test for effects of species, age, and their interaction using the R statistical language. Analysis of variance (ANOVA) tests with Tukey’s Honestly Significant Difference (HSD) were also conducted in R.

### Small molecule manipulation of Wnt signaling

Wnt signaling was chemically modulated starting at 5 dpf, a stage at which we detected differential expression of the Wnt pathway effectors *β*-*catenin* and *lef1* [30]. MZ and TT larvae were incubated with a Wnt agonist [44], lithium chloride (LiCl) (Sigma), to mimic the increased levels of Wnt signaling observed in LF. Three different treatments were conducted: (1) single treatment of 8 mM LiCl for 6 h at 5 dpf (following [30]), (2) long-term Wnt modulation with 8 mM LiCl for 6 h each at 5, 10, and 16 dpf, and (3) long-term Wnt modulation by continuous incubation in 250 μM LiCl (following [45]), replacing the LiCl solution every 2–4 days. Untreated larvae from the same brood were collected as controls. Following chemical treatment, the larvae were washed 3–5 times in system water, placed into a clean flask with fresh system water, and reared until the appropriate stage. Any dead fish (e.g., lockjaw phenotype [30]) were removed.

In a previous report, we also experimentally lowered Wnt signaling in the benthic foraging species, LF, using the chemical antagonist IWR-1 and were able to recapitulate a pelagic phenotype in larval fish [30]. Unfortunately, such an experiment could not be performed here. Mainly, this is because LF, the species in which a Wnt knockdown would be desirable, is highly sensitive to Wnt manipulation (e.g., increased frequency of lethal lockjaw phenotypes) [30]. This may reflect a more canalized Wnt signaling network in this phenotypically derived species, which results in a phenotype that is more robust to environmental changes but more sensitive to molecular changes [30]. Therefore, unlike other species, LF does not survive over long periods of development when treated with Wnt manipulators, rendering such experiments untenable.

## Results and discussion

### Overall patterns of ontogeny

All three aspects of craniofacial development—mandible, ventral, and lateral—share several notable characteristics. First, species-specific shapes can be detected at the earliest stage of development examined, which suggests that early developmental patterning events play an important role in determining adult morphology in cichlids. Second, the primary axis of shape variation for each aspect is ontogeny. Thus, differences between developmental stages exceed that between species. This finding suggests that all species, despite morphological differences in adults, share a common developmental trajectory. Third, within this common trajectory, there are species-specific developmental paths that differ in terms of orientation, size (path length), and/or shape. Taken together, cichlid craniofacial shapes appear to be determined early in development and elaborated via species-specific trajectories that represent variations on a common theme. We describe these patterns in greater detail below.

### Mandible development

**Fig. 3:**
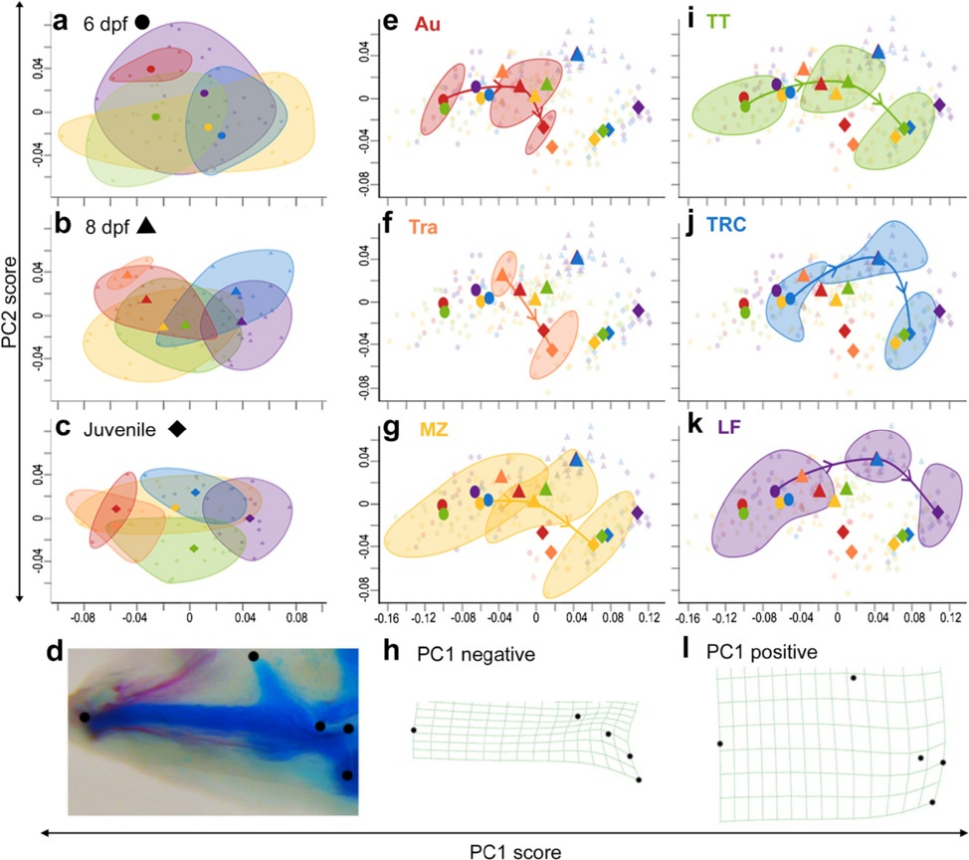
Mandible developmental trajectories are species-specific. Mandible morphospace is described by mandible length relative to depth of the processes (PC1) and the relative length of the retroarticular process (PC2). Differences in shape were analyzed either **a–c** within a single day or **e–g**, **i–k** through ontogeny. Species is indicated by *color* and time point is indicated by *symbol*, as designed in panel headings. *Ellipses* encompass all samples for the indicated species at a single time point, and mean PC scores are marked by large icon. Note that the plot is the same in (**e–g**, **i–k**), but with a different species highlighted. **d** Landmarks (*dots*) used in morphometric analysis. **h**, **l** Variation in mandible shape described by PC1 through ontogeny, depicted as deformation grids using thin-plate splines

Five LMs were used to describe variation in the shape of the mandible (Fig. 2b). After removing variation not due to shape (e.g., variation in size and orientation), data were analyzed using a PCA. The majority of shape variation (98.1 % TSV [total shape variation]) in the mandible through ontogeny is described by three principal axes: the relative length of the mandible relative to the processes, particularly the coronoid process (LM2, Fig. 2b) (PC1, 68.1 % TSV); the relative length of the retroarticular process (LMs 4–5 relative to LM3, Fig. 2b) (PC2, 12.9 % TSV); and the angle of the coronoid process to the mandible (PC3, 10.9 % TSV) (Fig. 3, [see Additional file 1: Figures S1 and S2]). MANOVA of PC1–3 scores shows a highly significant effect of species (Wilks’ *λ* = 0.56, *F*_5,197_ = 8.26, *p* < 0.0001), day (Wilks’ *λ* = 0.15, *F*_1,197_ = 357.45, *p* < 0.0001), and the species x day interaction (Wilks’ *λ* = 0.80, *F*_5,197_ = 3.00, *p* < 0.001) on mandibular shape.

Notably, the primary axis of variability (PC1) for each developmental stage is similar to the primary axis (PC1) of variation through ontogeny (see Additional file 1: Figure S2). Stated differently, the long/shallow and short/deep mandibular phenotypes that distinguish pelagic and benthic fish, respectively, at each stage (e.g., [see Additional file 1: Figure S3c and S3q]), also distinguish younger and older mandibles, respectively, within each species (e.g., [see Additional file 1: Figure S3a, c]). This pattern suggests that shape differences among species may be due to differences in the rate of development of this trait (more on this below). This pattern of variation also has important functional implications; all else being equal, a relatively long mandible gives pelagic fish faster jaw opening and closing, while a relatively short mandible should confer greater bite force for benthic fish to shear algae from rocks or crush hard prey [46]. Species differ significantly in mandible shape at all stages but become more distinct in shape through ontogeny (MANOVA of PC1–3 scores, 6 dpf: Wilks’ *λ* = 0.54, *F*_4,64_ = 3.61, *p* < 0.0001; 8 dpf: Wilks’ *λ* = 0.28, *F*_5,76_ = 7.98, *p* < 0.0001; juvenile: Wilks’ *λ* = 0.11, *F*_5,52_ = 11.58, *p* < 0.0001). Notably, at all developmental stages, the most extreme benthic species (LF) has a shorter mandible with deeper processes, while the most pelagic species (Au) has a longer mandible with more shallow processes (Fig. 3e–k [see Additional file 1: Figure S3r–t]).

At 6 dpf, all species occupy a similar region of morphospace (Fig. 3e–k) and have mandibles with relatively shallow processes (see Additional file 1: Figure S3a, f, i, l, o). Mandible development in all species then follows a common path, namely by extending the coronoid and retroarticular processes. However, species extend these processes to a different degree, producing significant species-specific differences in this trajectory (see Additional file 1: Figure S4). Even TRC and TT, species that have similar juvenile morphologies (Fig. 3) and feeding strategies (Table 1, [47]), have statistically distinct orientations in their developmental trajectories (see Additional file 1: Figure S4). This demonstrates that there are multiple developmental paths to produce similar phenotypes.

We also note a distinct heterochronic shift in mandible development between pelagic and benthic fish. Specifically, by 8 dpf, the extreme benthic species (LF) has developed a deeper coronoid process than the most pelagic species (Au) develops by juvenile stages (Fig. 3 and compare Additional file 1: Figure S3p and S3c). That is, Au retains a larval mandible phenotype, consistent with paedomorphism. This observed difference in the rate of development of mandibular shape is reflected in the statistical differences in trajectory size between species (see Additional file 1: Figure S4) and underscores the importance of heterochrony in promoting species-specific morphologies.

### Ventral development

**Fig. 4:**
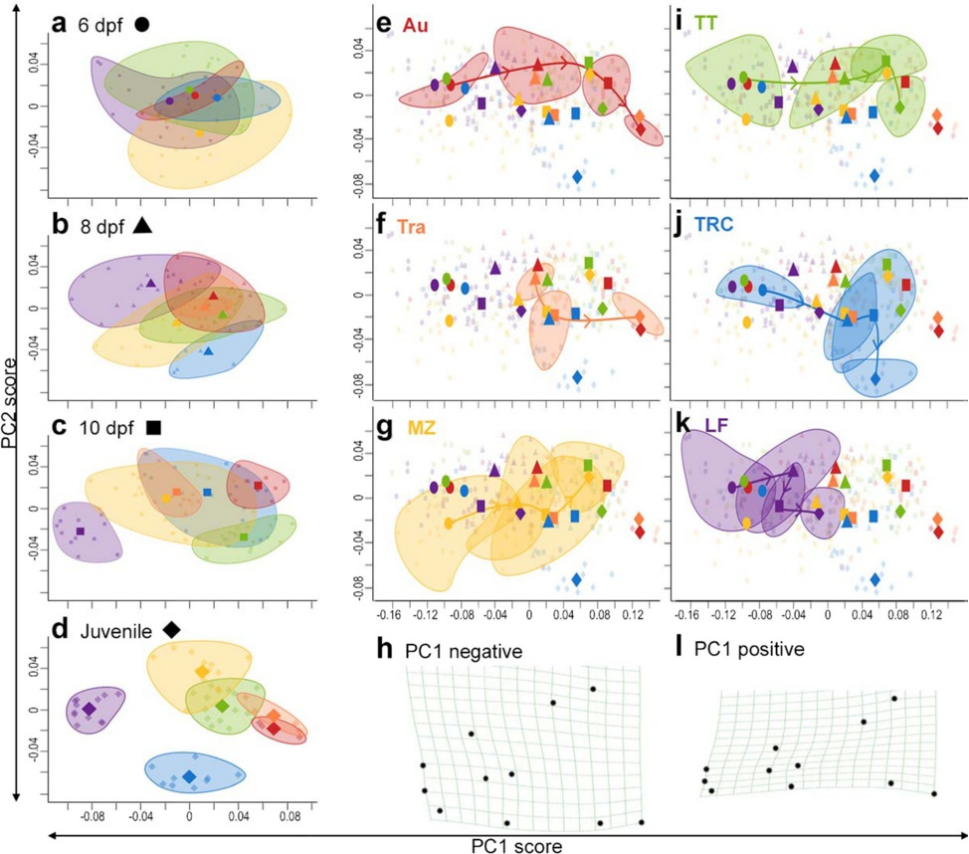
Ventral developmental trajectories are species-specific. Ventral morphospace is described by relative jaw width and mandible length (PC1) and distance between the basihyal to the mandible (PC2). Differences in shape were analyzed either **a–d** within a single day or **e–g**, **i–k** through ontogeny. Colors, symbols, and ellipses are as described in Fig. 3. Note that the plot is the same in (**e–g**, **i–k**), but with a different species highlighted. **h**, **l** Variation in ventral shape described by PC1 through ontogeny, depicted as deformation grids using thin-plate splines

Eleven LMs were used to describe variation in shape for the ventral aspect of the pharyngeal skeleton, including the mandible, hyoid and ceratobranchial structures (Fig. 2c). The majority of shape variation (82.3 % TSV) in ventral ontogeny is captured by three principal axes (Fig. 4 [see Additional file 1: Figures S5 and S6]). The first axis (PC1, 63.2 % TSV) describes width of the mandible and pharyngeal skeleton (distance between LMs 2–3, LMs 4–5, LMs 6–7, and LMs 8–9, Fig. 2c) as well as mandible length (distance from LM1 to LM4 and LM1 to LM5, Fig. 2c). The second axis (PC2, 10.7 % TSV) also includes some aspects of width but primarily distinguishes the distance of the basihyal (LMs 10–11, Fig. 2c) to the mandible (LM1). The third axis (PC3, 8.4 % TSV) distinguishes a wider, more trapezoid shaped mandible (LMs 1–5, Fig. 2c) versus a narrower, more triangular mandible shape (see Additional file 1: Figure S6). MANOVA of PC1–3 scores shows a highly significant effect of species (Wilks’ *λ* = 0.18, *F*_5,275_ = 42.70, *p* < 0.0001), day (Wilks’ *λ* = 0.20, *F*_1,275_ = 357.25, *p* < 0.0001), and the species × day interaction (Wilks’ *λ* = 0.59, *F*_5,275_ = 10.77, *p* < 0.0001) on ventral shape.

As with mandibular development, the primary axis of variation in ventral shape (i.e., width) at each individual developmental time point is the same axis of variation through ontogeny (see Additional file 1: Figure S6), which again points toward developmental rate as an important factor in determining adult shape difference. Species differ significantly in ventral shape at all stages of development, becoming increasingly distinct as development progresses (MANOVA of PC1–3 scores, 6 dpf: Wilks’ *λ* = 0.25, *F*_4,60_ = 8.89, *p* < 0.0001; 8 dpf: Wilks’ *λ* = 0.13, *F*_5,74_ = 14.66, *p* < 0.0001; 10 dpf: Wilks’ *λ* = 0.036, *F*_5,79_ = 33.07, *p* < 0.0001; juvenile: Wilks’ *λ* = 0.0013, *F*_5,51_ = 92.49, *p* < 0.0001). At 10 dpf and juvenile stages, this is driven largely by shape variation in the most benthic species (LF), which occupies a distinct, non-overlapping aspect of shape space characterized by a wide pharyngeal skeleton and short mandible (see Additional file 1: Figure S7w). Meanwhile, pelagic fish develop relatively long, narrow mandibles (LMs 2–5) that flare out to a wider branchial region (LMs 6–9) (Fig. 2c, [see Additional file 1: Figure S7d, g, k]). This triangular shape of the pharyngeal skeleton, and hence the buccal cavity, in pelagic fish should allow for a higher flow velocity during suction feeding [48].

All species have relatively wide and short jaws at 6 dpf (see Additional file 1: Figure S7a, h, l, p, t), which narrow and lengthen over time. While all species share this general ontogenetic trajectory, there are also species-specific paths (see Additional file 1: Figure S4), including evidence of heterochrony. Specifically, by 8 dpf, the pharyngeal skeleton of the most pelagic species (Au) is narrower than that of the most benthic species (LF) at juvenile stages (Fig. 4 and compare Additional file 1: Figure S7b and w). In other words, LF retains a larval ventral phenotype well into juvenile stages of development and may be paedomorphic in terms of ventral shape (again, see highly significant differences in trajectory size between LF and Au) (see Additional file 1: Figure S4). Notably, this observation is opposite to what was observed for the mandible in the lateral view, where LF exhibited accelerated development relative to other species. These opposing trends underscore the idea that different aspects of the facial skeleton develop and evolve independently to one another (i.e., are modular) even though they have considerable functional interactions [49] (see Conclusions).

### Lateral development

**Fig. 5:**
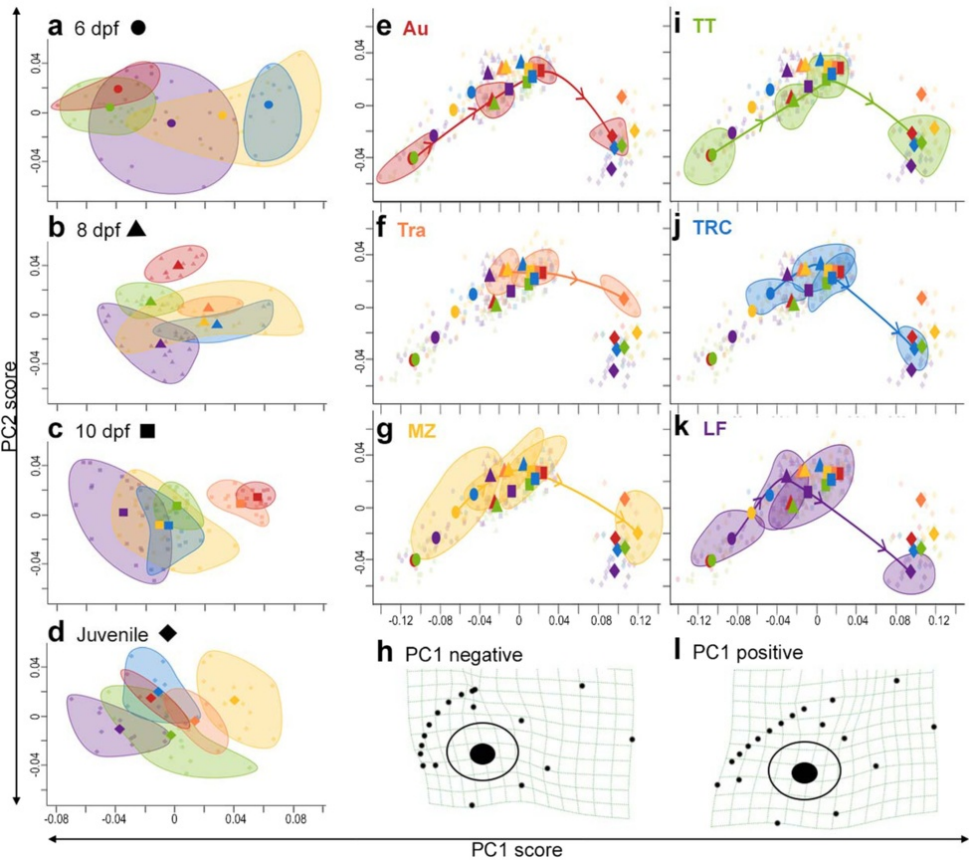
Lateral developmental trajectories are species-specific. Lateral morphospace is predominantly described by degree of facial outgrowth, particularly the pre-orbital region, and commensurate changes in craniofacial slope (PC1) as well as the relative proportions of the pre-orbital region and neurocranium, again with commensurate changes in craniofacial slope (PC2). Differences in shape were analyzed either **a–d** within a single day or **e–g, i–k** through ontogeny. Colors, symbols, and ellipses are as described in Fig. 3. Note that the plot is the same in **(e–g, i–k)**, but with a different species highlighted. **h, l** Variation in lateral shape described by PC1 through ontogeny, depicted as deformation grids using thin-plate splines

The lateral aspect of the craniofacial skeleton encompasses distinct developmental, anatomical, and functional modules, including the primarily neural crest cell-derived pre-orbital (i.e., facial) region and the primarily mesoderm-derived neurocranium [17]. The lateral skeleton, including the brain case, was characterized using ten landmarks. We also utilized eight evenly spaced semilandmarks to define the slope of the pre-orbital region (Fig. 2a), differences in which have been shown in both cichlids [49] and finches [50] to impact bite force. Three primary axes accounted for 78.9 % TSV in lateral ontogeny. The primary axis of variation (PC1, 58.9 % TSV) describes differences in facial outgrowth, particularly the pre-orbital region (distance between LMs 1–2 and LMs 3, 5, 8, and 9, Fig. 2a), and commensurate changes in the craniofacial slope. Additional shape variation is due to differences in the relative proportions of the facial region (LMs 1–5 and 8–10, Fig. 2a) and the neurocranium (LMs 5–8, Fig. 2a), again with commensurate changes in the craniofacial slope (PC2, 10.8 % TSV), and outgrowth in the posterior region of the neurocranium (particularly LM6, Fig. 2a) (PC3, 10.1 % TSV) (Fig. 5, [see Additional file 1: Figures S4, S8–S10]). There is a highly significant effect of species (Wilks’ *λ* = 0.49, *F*_5,286_ = 15.20, *p* < 0.0001), day (Wilks’ *λ* = 0.043, *F*_1,286_ = 2130.96, *p* < 0.0001), and the species x day interaction (Wilks’ *λ* = 0.78, *F*_5,286_ = 4.79, *p* < 0.0001) on lateral shape.

As with mandibular and ventral shape development, the primary axis of variation in lateral shape (i.e., craniofacial slope) at each individual stage is similar to the primary axis of variation through ontogeny (see Additional file 1: Figure S9). This is consistent with the hypothesis that shape differences among species are due to changes in timing and/or rate of ontogeny. The species are significantly different in lateral shape at all stages of development (MANOVA of PC1–3 scores, 6 dpf: Wilks’ *λ* = 0.25, *F*_4,62_ = 9.21, *p* < 0.0001; 8 dpf: Wilks’ *λ* = 0.093, *F*_5,73_ = 17.91, *p* < 0.0001; 10 dpf: Wilks’ *λ* = 0.085, *F*_5,89_ = 23.17, *p* < 0.0001; juvenile: Wilks’ *λ* = 0.052, *F*_5,51_ = 17.33, *p* < 0.0001). Based on the MANOVA results, the species are most distinct at 10 dpf (compare overlap of ellipses in Fig. 5c to Fig. 5a–b), and this is primarily due to divergence in shape between sand-dwelling pelagic fish (Au and Tra) and rock-dwelling benthic fish (MZ, TT, TRC, and LF). Notably, by juvenile stages, the most pelagic rock-dwelling species (MZ) occupies the most extreme position along PC1, which is consistent with shifts in species-specific rates of development. However, unlike mandibular and ventral shapes, we do not detect obvious instances of heterochrony in the lateral view (Fig. 5, [see Additional file 1: Figure S4]). Development in the lateral view is also distinct in that whereas the ontogenetic morphospace for mandible and ventral development is largely continuous across stages (e.g., Figs. 3e and 4e), lateral shape at juvenile stages occupies a distinct region of the morphospace compared to larval stages. This suggests that a large degree of craniofacial development occurs in the lateral skeleton between 10 dpf and juvenile stages (approximately 1 month).

### Early manipulation of Wnt signaling results in modest shifts in larval morphology

With an appreciation for some of the similarities and differences in normal craniofacial development among cichlid species (Figs. 3, 4 and 5), we next sought to determine the extent to which molecular manipulations could affect species-specific developmental trajectories. Namely, we wanted to ask if manipulation of a major signal transduction pathway during early craniofacial development was sufficient to shift a developmental trajectory into morphospace normally occupied by another species. We have previously shown that activity of the Wnt signaling network is correlated with changes in craniofacial slope [30], the primary variation observed in the current analysis of lateral development (Fig. 5). Specifically, the obligate benthic species LF demonstrates increased levels of Wnt signaling relative to more pelagic fish at the onset of craniofacial bone development (i.e., 5–6 dpf) [30]. Further, artificial up-regulation of Wnt activity in a pelagic fish at 5 dpf resulted in a more benthic phenotype at 8 dpf [30]. However, what remains unknown is whether the effect of such molecular modulation is limited to short-term shifts in shape, or if these shifts will be maintained over longer periods of development. Therefore, we artificially increased levels of Wnt signaling with the small molecule agonist LiCl (which up-regulates that pathway through inhibition of GSK3 [44]) in species toward the pelagic end of the spectrum (MZ and TT) to mimic the increased Wnt activity observed in the most benthic species, LF [30].

**Fig. 6:**
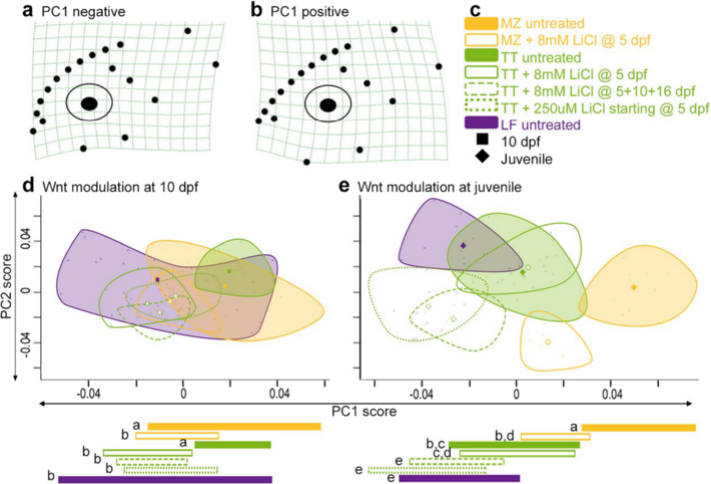
Continued modulation of Wnt signaling shifts lateral developmental trajectory. **a**, **b** Variation in lateral shape described by PC1 at juvenile stages following Wnt modulation. PC1 at 10 dpf describes similar variation (see Additional file 1: Figure S9). **c** Legend of colors and symbols used. **d** Lateral morphospace at 10 dpf. **e** Lateral morphospace at juvenile stages. *Bars* underneath plots indicate range of PC1 scores for each species/treatment. *Letters* by bars indicate statistical grouping based on ANOVA with Tukey’s HSD using a cutoff of *p* = 0.05

Wnt signaling was induced by incubating the larvae in 8 mM LiCl for 6 h at 5 dpf, a stage at which we detected differential expression of the Wnt pathway effectors *β*-*catenin* and *lef1* [30]. Resultant phenotypic effects (Fig. 6, [see Additional file 1: Figures S4 and S11]) were assessed through the time course of development described above (Fig. 1). As expected, by 10 dpf, the more pelagic species MZ and TT treated with LiCl developed lateral craniofacial skeletons that were statistically indistinguishable from that of LF, effectively phenocopying the benthic eco-type (Fig. 6d, *p* = 0.969 for MZ with LiCl versus LF; *p* = 0.977 for TT with LiCl versus LF, ANOVA with Tukey’s HSD of PC1 scores). However, while this early change in molecular signaling shifted a developmental trajectory into morphospace normally occupied by another species at 10 dpf, craniofacial shape recovers by juvenile stages (Fig. 6e). Specifically, juvenile TT treated with 8 mM LiCl for 6 h at 5 dpf do not differ in lateral shape compared to the untreated TT samples (*p* = 0.999). MZ treated with LiCl still possess a significantly steeper (i.e., more benthic) craniofacial profile relative to the untreated MZ samples (*p* < 0.0001) and phenocopy untreated TT samples (*p* = 0.438) at juvenile stages (*p* values from ANOVA with Tukey’s HSD of PC1 scores). However, lateral shape has recovered relative to LF. In other words, early and relatively brief modulation of Wnt signaling is sufficient to shift an MZ developmental trajectory to that normally occupied by another species (TT). However, this short-term treatment is insufficient to result in the extreme benthic morphology of LF [36].

### Sustained modulation of Wnt signaling is sufficient for extreme and sustained shifts in craniofacial morphology

Given that an early change in development was not sufficient to develop the extreme, derived morphology of the most benthic species, LF [36] (Fig. 6e), we hypothesized that signaling changes might need to be reinforced throughout development. This makes sense within the context of Wnt signaling and bone development, as this pathway regulates bone cell differentiation [28], and our previous work in cichlids and zebrafish suggests that higher levels of Wnt signaling mediate shifts in craniofacial shape by accelerating rates of bone deposition [30]. Relative to the mandible and the ventral pharyngeal skeleton, the lateral aspect of the craniofacial skeleton undergoes significant amounts of bone development between larval and juvenile stages (Fig. 1). Indeed, very little of the cranium or upper jaw apparatus have begun to mineralize at 10 dpf. We therefore hypothesized that sustained Wnt modulation is required to affect craniofacial shape through early juvenile stages, and specifically to shift the TT developmental trajectory to that of the most benthic species, LF.

In order to test this hypothesis, we altered Wnt signaling over extended periods of development in TT by either (1) incubating the larvae in 8 mM LiCl for 6 h each at 5, 10, and 16 dpf or (2) continuously raising the larvae in 250 μM LiCl (following [45]). Not only did these treatments in TT phenocopy an LF-like morphology at 10 dpf (Fig. 6d, *p* = 0.800 for TT with 250 μM LiCl versus LF; *p* = 0.999 for TT with 8 mM LiCl at 5,10, and 16 dpf versus LF, ANOVA with Tukey’s HSD of PC1 scores), but they also resulted in fish that retained a benthic phenotype at juvenile stages (Fig. 6e). Specifically, the TT samples with sustained modulation of Wnt signaling (either continuously treated with 250 μM LiCl or treated with pulses of 8 mM LiCl at 5, 10, and 16 dpf) possessed juvenile craniofacial shapes that were statistically indistinguishable from LF along PC1, which describes variation in the craniofacial slope (*p* = 0.115 for TT with 250 μM LiCl versus LF; *p* = 0.992 for TT with 8 mM LiCl at 5, 10, and 16 dpf versus LF, ANOVA with Tukey’s HSD). Overall, these data suggest that the evolution of progressively more extreme craniofacial morphologies may have occurred through sustained alterations in molecular signaling over extended periods of craniofacial skeletal development.

### Modulation of Wnt signaling does not affect other aspects of craniofacial shape in a predictable fashion

LiCl treatment in pelagic species did not result in the development of a benthic-like mandible or ventral phenotypes (see Additional file 1: Figure S11). This is not to say that Wnt signaling is not involved in the development of species-specific mandibular or ventral shapes. The possibility remains that it does, but at an earlier stage in development. Nevertheless, upon treatment with LiCl, the mandibular, ventral, and lateral aspects of the craniofacial skeleton are not responding in a coordinated manner. This observation underscores the modular nature of craniofacial development (see Conclusions) and is consistent with the hypothesis that adult craniofacial shape is a compilation of variation in multiple molecular and developmental pathways that act during distinct periods of time.

## Conclusions

### Variations on a theme: species-specific phenotypes within a conserved developmental program

Overall, we find that for all three aspects of craniofacial development—mandibular, lateral, and ventral—species have a similar path of ontogeny, despite drastic differences in adult morphologies. This conservation of ontogenetic trajectories suggests that developmental processes constrain craniofacial evolution in cichlids. This is similar to what was previously observed in amniotes, where deviations from the developmental bauplan resulted in maladaptive facial clefting [11]. While teleosts lack the conspicuous facial prominences that characterize early craniofacial development in amniotes, their development also requires the coordinated outgrowth and fusion of distinct skeletal elements in the head. Thus, the conserved pattern of craniofacial development we observe in cichlids (Figs. 3, 4 and 5) may similarly be the result of selection against non-viable facial malformations. However, we also have evidence that alterations within this common developmental program can produce phenotypic variation. We detect significant species-specific differences in the timing (e.g., heterochrony) and/or direction of developmental trajectories, indicating that both specific (e.g., alteration of Wnt signaling during bone development) and general (e.g., heterochrony) changes in development contribute to phenotypic variation.

### The importance of ontogeny in producing phenotypic variation

A common, often implicit, assumption in evolutionary developmental biology (evo-devo) is that phenotypic variation is the result of early developmental changes that are elaborated to produce adult morphologies. However, here, we demonstrate that craniofacial development is robust and such early changes may not be sufficient to elicit long-lasting effects—i.e., that phenotypic changes may be compensated by later ontogenetic events. In particular, while early, short-term modulation of Wnt signaling produced phenotypic variation in larval stages, these were largely counteracted by juvenile stages. This observation underscores the importance of post-embryonic development in evolutionary change [51]. Further, it emphasizes that adult morphologies are the result of many molecular changes that act over extended periods of development [13] and on distinct tissues, for instance *lbh* during neural crest cell development [37] and Wnt signaling during bone deposition [30]. A significant future challenge facing practitioners of evo-devo is to reconstruct the totality of molecular and developmental shifts that result in species-specific adult morphologies.

### Modularity in craniofacial development and evolution

Two lines of evidence suggest that mandibular, ventral, and lateral skeletal structures are independent developmental modules within the craniofacial complex. The first involves patterns of developmental rate. Juvenile individuals of the most pelagic species (Au) retain larval mandibular phenotypes but exhibit accelerated rates of development with respect to the ventral pharyngeal skeleton. On the other hand, the extreme benthic species (LF) demonstrates the opposite pattern and retains a larval phenotype as juveniles for the ventral view but exhibits accelerated rates of mandibular development. In other words, the mandibular and ventral skeletons are developmentally independent and a single species does not simply retain the larval phenotype for all aspects of craniofacial skeleton. Second, modulation of Wnt signaling has a significant and largely predictable impact on lateral craniofacial development (e.g., craniofacial slope), but not on the mandible or ventral pharyngeal skeleton. This is similar to what has been proposed in finches [52–54], where changes in different signaling pathways independently regulate different dimensions of the beak.

This pattern of modularity should have important influences on the direction and/or speed of evolutionary change (i.e., evolvability) [3, 55–60]. A significant challenge of future research will be to determine how the de-coupling of mandibular, ventral, and lateral development in the cichlid craniofacial skeleton has impacted evolutionary constraints in this group. For instance, it has been proposed [61] that new patterns of modularity may contribute to the extensive morphological adaptations in this lineage, effectively serving as a “key innovation” [62, 63]. Overall, this work demonstrates the dual role of development in promoting and constraining phenotypic variation and illustrates the role of ontogeny in the evolution of complex shapes [13, 14].

## Additional file

Additional file 1: Table S1. Sample number and feeding strategies for species used in morphometric analyses. **Figure S1.** Mandible morphospace for PC1 and PC3. **Figure S2.** Deformation warps for mandible development. **Figure S3.** Species-specific mandible development. **Figure S4.** Developmental trajectories have species-specific paths. **Figure S5.** Ventral morphospace for PC1 and PC3. **Figure S6.** Deformation warps for ventral development. **Figure S7.** Species-specific ventral development. **Figure S8.** Lateral morphospace for PC1 and PC3. **Figure S9.** Deformation warps for lateral development. **Figure S10.** Species-specific lateral development. **Figure S11.** Wnt modulation does not alter ventral or mandible developmental trajectories.

## Abbreviations

ANOVA: Analysis of variance
Au: *Aulonocara* sp
hpf: Hours post fertilization
LF: *Labeotropheus fuelleborni*
LiCl: Lithium chloride
LM: Landmark
MANOVA: Multivariate analysis of variance
MZ: *Maylandia zebra*
PC: Principal component
PCA: Principal component analysis
sp.: Species
Tra: *Tramitichromis* sp.
TRC: *Tropheops* sp. “red cheek”
TSV: Total shape variation
TT: *Tropheops tropheops*
Tukey’s HSD: Tukey’s Honestly Significant Difference
