# Supplementary material for: Constraint and diversification of developmental trajectories in cichlid facial morphologies
Source: EvoDevo. 2015 Jun 28;6:25. doi: 10.1186/s13227-015-0020-8 (PMC4518560; doi:10.1186/s13227-015-0020-8)
Supplement: Additional file 1: Table S1. — Sample number and feeding strategies for species used in morphometric analyses. Figure S1. Mandible morphospace for PC1 and PC3. Figure S2. Deformation warps for mandible development. Figure S3. Species-specific mandible development. Figure S4. Developmental trajectories have species-specific paths. Figure S5. Ventral morphospace for PC1 and PC3. Figure S6. Deformation warps for ventral development. Figure S7. Species-specific ventral development. Figure S8. Lateral morphospace for PC1 and PC3. Figure S9. Deformation warps for lateral development. Figure S10. Species-specific lateral development. Figure S11. Wnt modulation does not alter ventral or mandible developmental trajectories. [file 13227_2015_20_MOESM1_ESM.pdf]

Species (and treatment, as applicable)	Habitat	Feeding Strategy	Color in figures	6dpf	8dpf	10dpf	juvenile
Aulonocara sp. (Au)	sand	sonar hunting		n= 4 fam=1	n=12 fam=2	n=10 fam=1	n=3 fam=1
Tramitichromis sp. (Tra)	sand	sifting		n=0 fam=0	n=3 fam=1	n=10 fam=2	n=4 fam=1
Maylandia zebra (MZ)	rock	suction/ combing		n=18 fam=6	n=18 fam=5	n=20 fam=5	n=15 fam=1
Tropheops tropheops (TT)	rock	biting: nip & twist		n=20 fam=5	n=20 fam=6	n=20 fam=5	n=14 fam=2
Tropheops sp. “”red cheek” (TRC)	rock	biting: nip & twist		n=8 fam=2	n=7 fam=3	n=15 fam=4	n=9 fam=1
Labeotropheus fuelleborni (LF)	rock	biting: scraping		n=17 fam=5	n=20 fam=5	n=20 fam=4	n=12 fam=1
Maylandia zebra 8mM LiCl, 6hr @ 5 dpf				n=12 fam=3	n=16 fam=3	n=13 fam=3	n=11 fam=3
Tropheops tropheops 8mM LiCl, 6hr @ 5 dpf				n=21 fam=3	n=21 fam=3	n=21 fam=3	n=15 fam=3
Tropheops tropheops 8mM LiCl, 6hr @ 5,10,16 dpf				n=9 fam=2	n=11 fam=2	n=10 fam=2	n=10 fam=2
Tropheops tropheops 250uM LiCl, continuously starting @ 5 dpf				n=15 fam=3	n=17 fam=3	n=16 fam=3	n=12 fam=3

Table S1: Sample number and feeding strategies for species used in morphometric analyses. Specimens were evenly sampled from all families (fam).

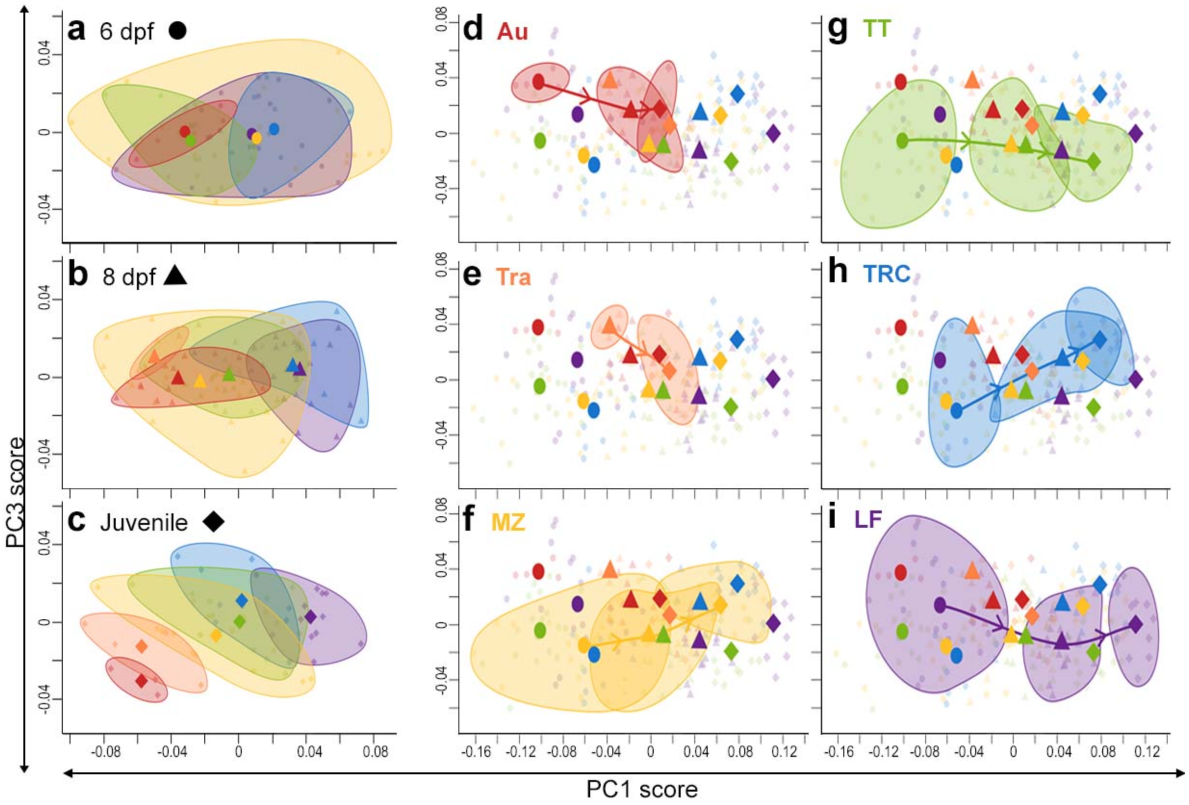

Figure S1. Mandible morphospace for PC1 and PC3. Differences in mandible shape are described by described by relative mandible length relative to depth of the processes (PC1) and the angle of the coronoid process to the mandible (PC3). Differences were analyzed both **(a-c)** within a single day and **(d-i)** over ontogeny. Colors, symbols, and ellipses are as described in Figure 3.

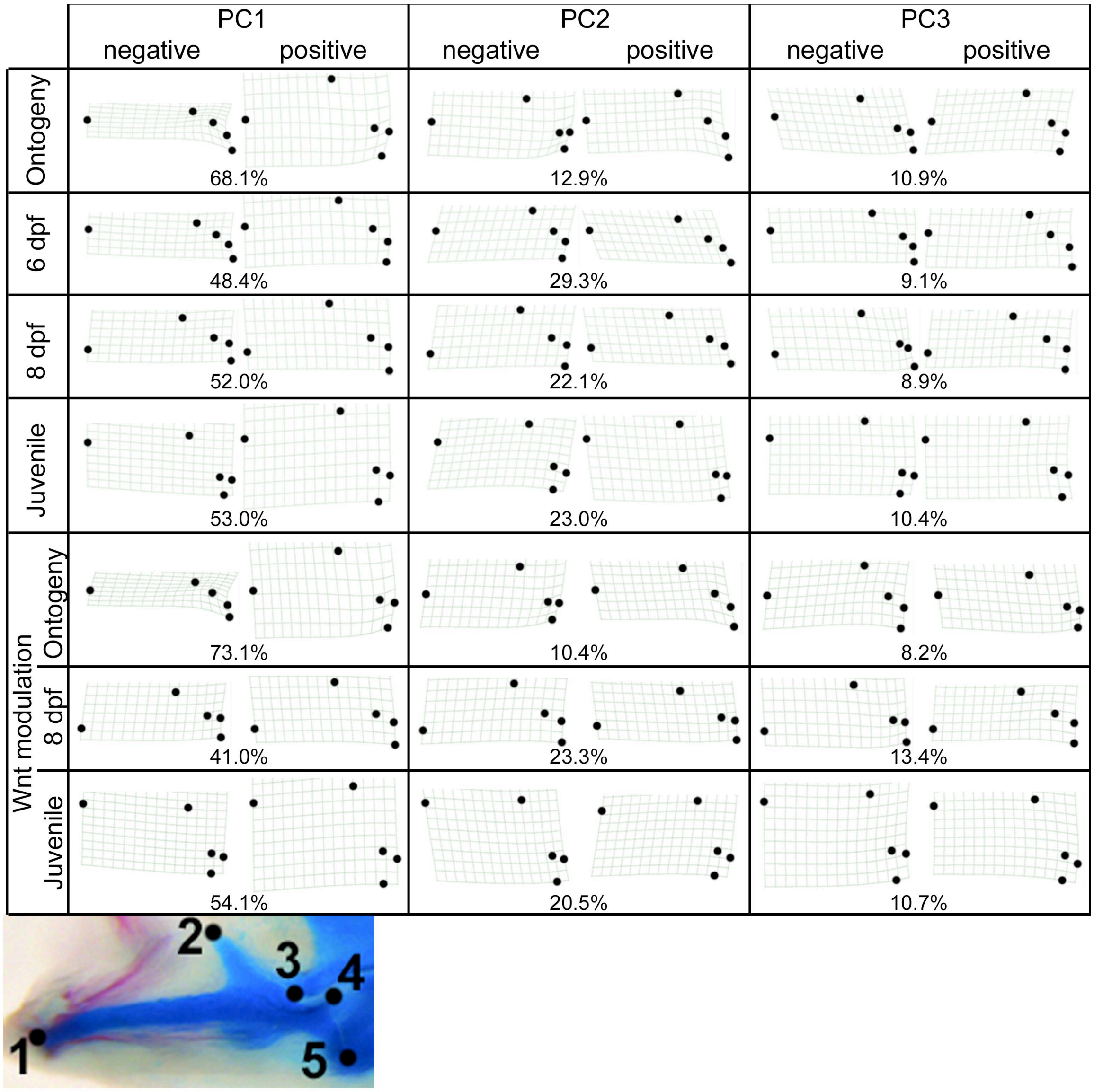

Figure S2. Deformation warps for mandible development. Visualization of mandible shape changes and percent of total shape variation described by principal components 1-3, analyzed both within a single day and through ontogeny.

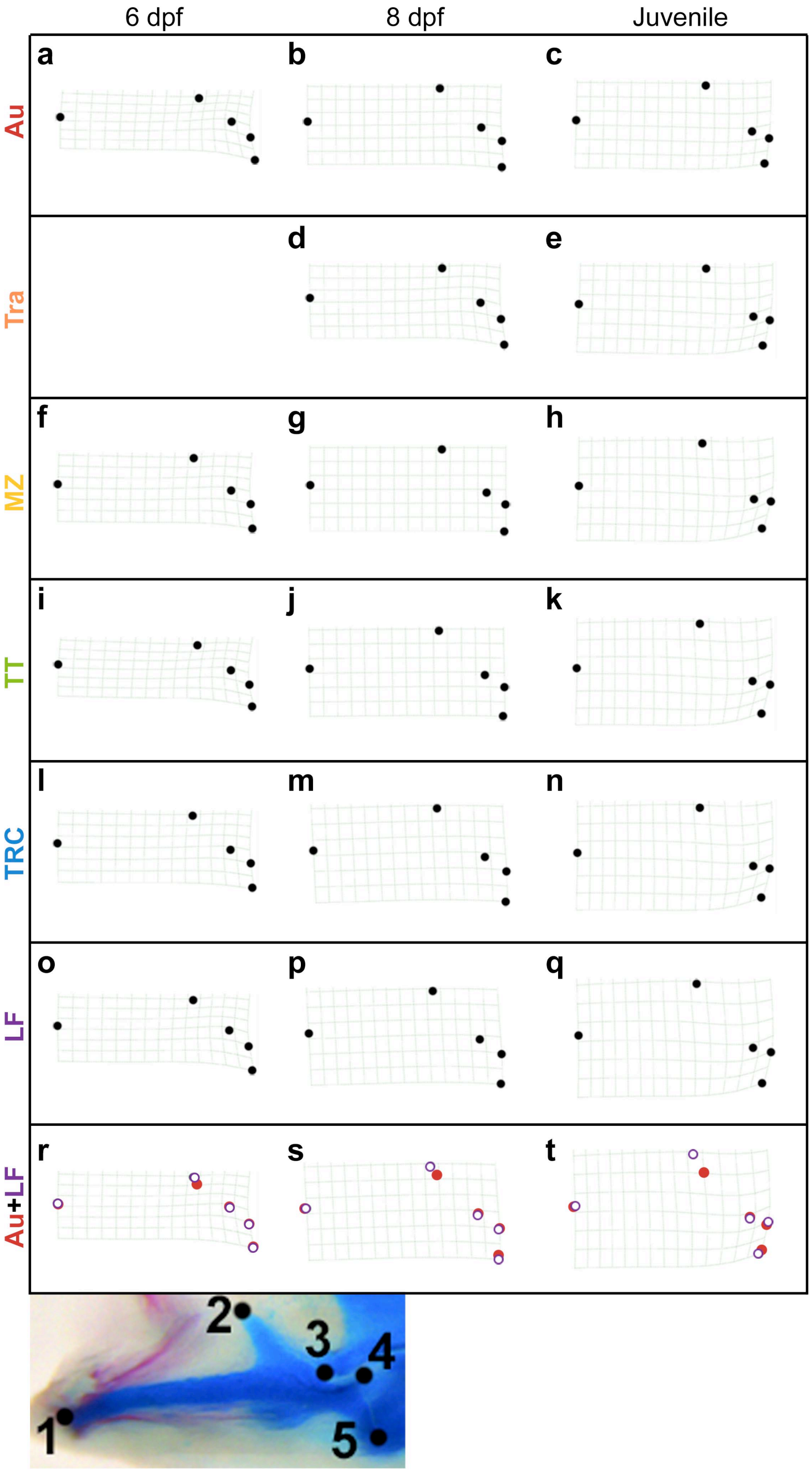

Figure S3. Species-specific mandible development. Deformation grids representing mean PC1 and mean PC2 score for each species at each developmental timepoint: **(a-c)** Au, **(d-e)** Tra, **(f-h)** MZ, **(i-k)** TT, **(l-n)** TRC, and **(o-q)** LF. **(r-t)** Overlay of Au in red and LF in purple at each developmental stage.

a Mandible Trajectory

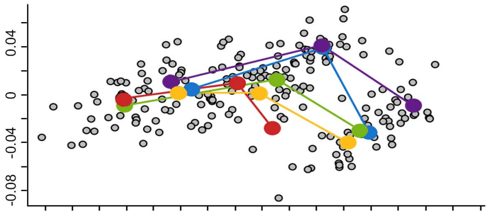

b

	Au	MZ	TT	TRC	LF
Au					
MZ	O Sh				
TT	S	O S Sh			
TRC	O S	O S Sh	O		
LF	S	O S Sh		O	

c Ventral Trajectory

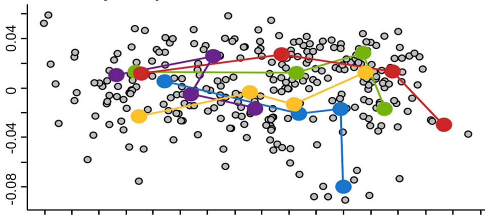

d

	Au	MZ	TT	TRC	LF
Au					
MZ	O S				
TT		O S Sh			
TRC	O Sh	O Sh	O Sh		
LF	O S Sh	O Sh	O S Sh	O S Sh	

e Lateral Trajectory

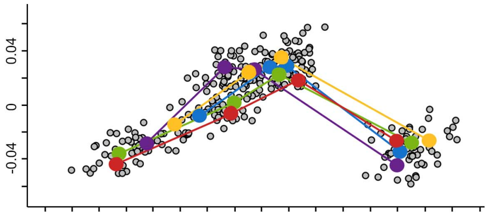

f

	Au	MZ	TT	TRC	LF
Au					
MZ	O Sh				
TT		O Sh			
TRC	O Sh	O	O Sh		
LF	O Sh	O	O Sh	O	

g Mandible with LiCl treatments Trajectory

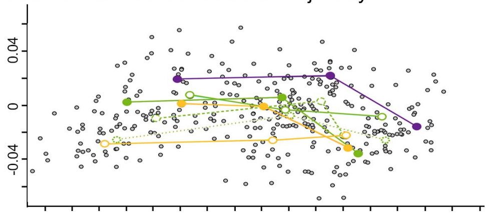

h

	LF	MZ	MZ + 8mM LiCl @ 5 dpf	TT	TT + 250uM LiCl starting @ 5 dpf	TT + 8mM LiCl @ 5+10+16 dpf	TT + 8mM LiCl @ 5 dpf
LF							
MZ	O S Sh						
MZ + 8mM LiCl @ 5 dpf	O	O S Sh					
TT		O S Sh	O				
TT + 250uM LiCl starting @ 5 dpf	O S Sh	O	O S Sh	O S			
TT + 8mM LiCl @ 5+10+16 dpf	O S Sh	O Sh	O	O Sh	Sh		
TT + 8mM LiCl @ 5 dpf	O	O S Sh		O	S	S Sh	

i Ventral with LiCl treatments Trajectory

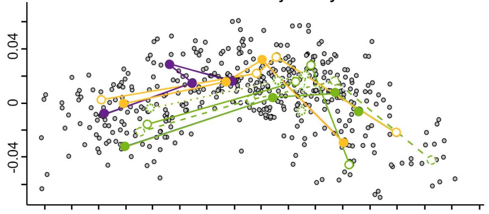

j

	LF	MZ	MZ + 8mM LiCl @ 5 dpf	TT	TT + 250uM LiCl starting @ 5 dpf	TT + 8mM LiCl @ 5+10+16 dpf	TT + 8mM LiCl @ 5 dpf
LF							
MZ	O Sh						
MZ + 8mM LiCl @ 5 dpf	O S Sh	O S					
TT	O S Sh	O S Sh	O Sh				
TT + 250uM LiCl starting @ 5 dpf	O S Sh	O S Sh	O Sh	O Sh			
TT + 8mM LiCl @ 5+10+16 dpf	O S Sh	O S	O	O Sh	O Sh		
TT + 8mM LiCl @ 5 dpf	O Sh	O Sh	O S Sh	O S	O S	O S Sh	

k Lateral with LiCl treatments Trajectory

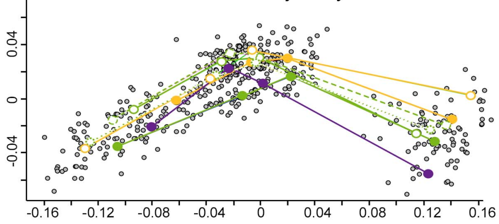

l

	LF	MZ	MZ + 8mM LiCl @ 5 dpf	TT	TT + 250uM LiCl starting @ 5 dpf	TT + 8mM LiCl @ 5+10+16 dpf	TT + 8mM LiCl @ 5 dpf
LF							
MZ	O						
MZ + 8mM LiCl @ 5 dpf	O S Sh	O S Sh					
TT	O Sh	O Sh	O S				
TT + 250uM LiCl starting @ 5 dpf	O S	O Sh	O S	O Sh			
TT + 8mM LiCl @ 5+10+16 dpf	O Sh	O S Sh	O	O Sh	O S		
TT + 8mM LiCl @ 5 dpf	O S Sh	O S Sh	O	O S	O S Sh	O	

Figure S4. Developmental trajectories have species-specific paths. Analysis of orientation (O), size (i.e. path length, S), and shape (Sh) of **(a-b, g-h)** mandible, **(c-d, i-j)** ventral, and **(e-f, k-l)** lateral development normally and following Wnt modulation, respectively. **(b,d,f,h,j,l)** Summary of statistical significance based on 10,000 residual randomization permutations. Gray indicates p<0.05 and black indicates p<0.005 (Bonferroni corrected).

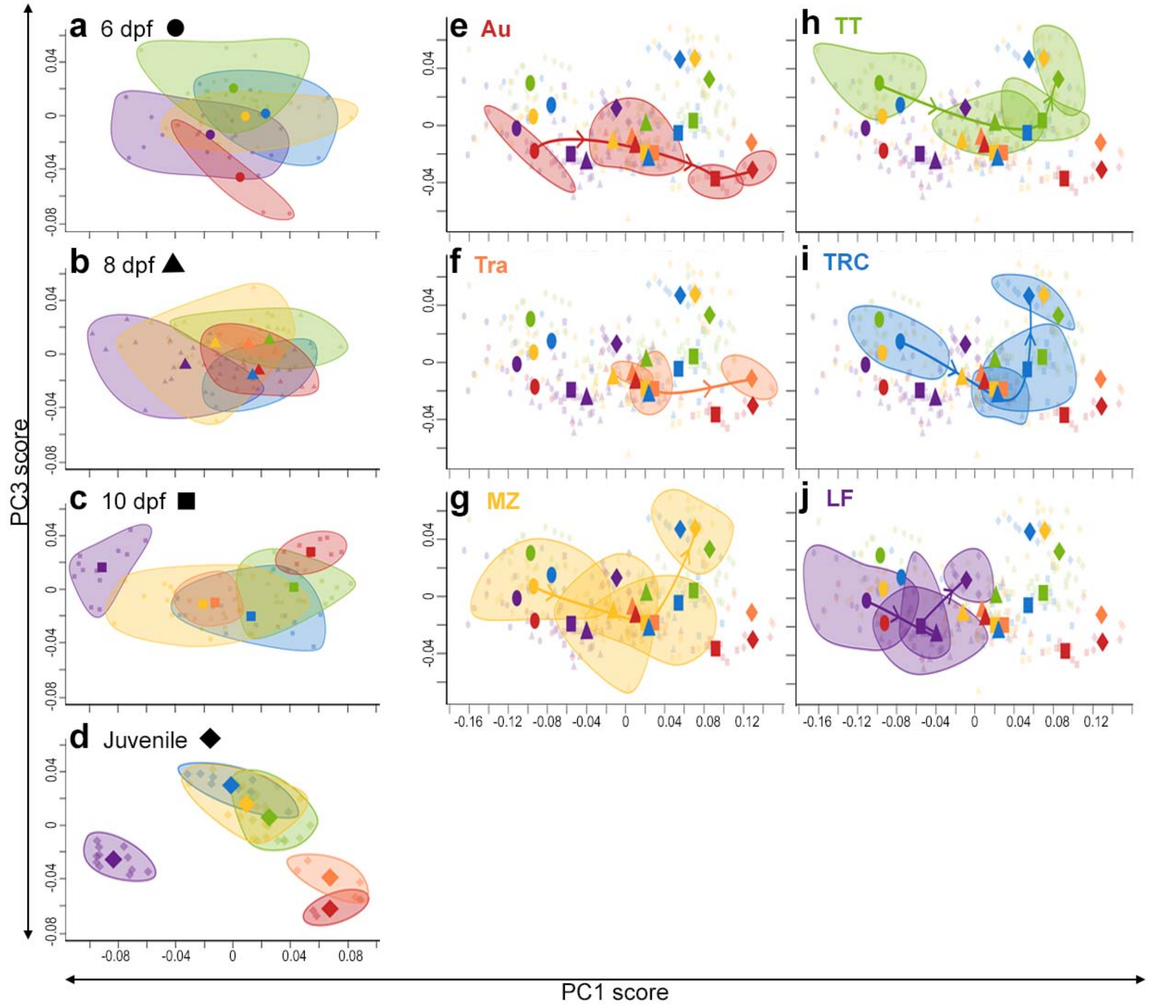

Figure S5. Ventral morphospace for PC1 and PC3. Differences in ventral shape are described by described by relative jaw width and mandible length (PC1) and the width of the mandible (PC3). Differences were analyzed both **(a-c)** within a single day and **(d-i)** over ontogeny. Colors, symbols, and ellipses are as described in Figure 3.

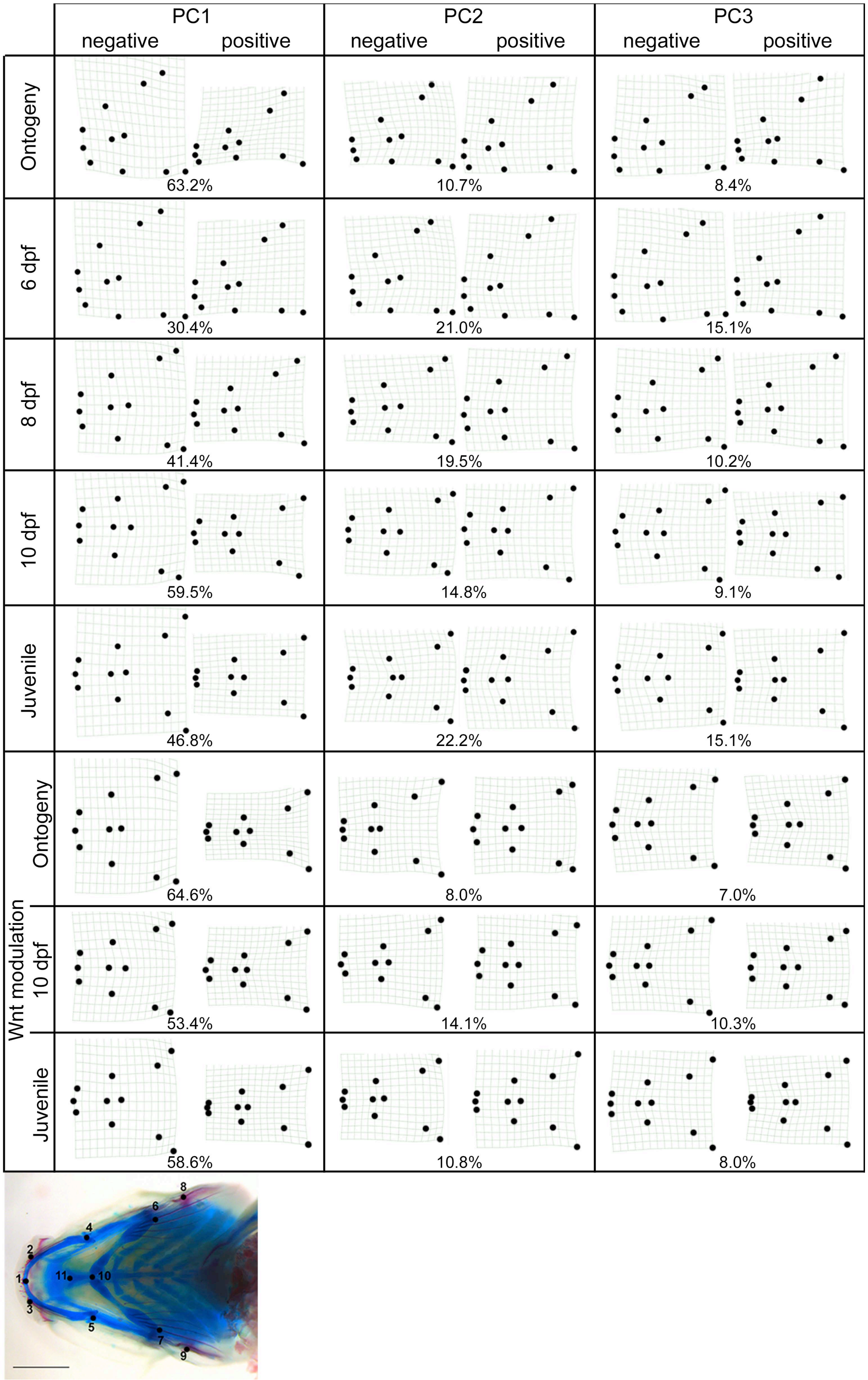

Figure S6. Deformation warps for ventral development. Visualization of ventral shape changes and percent of total shape variation described by principal components 1-3, analyzed both within a single day and through ontogeny.

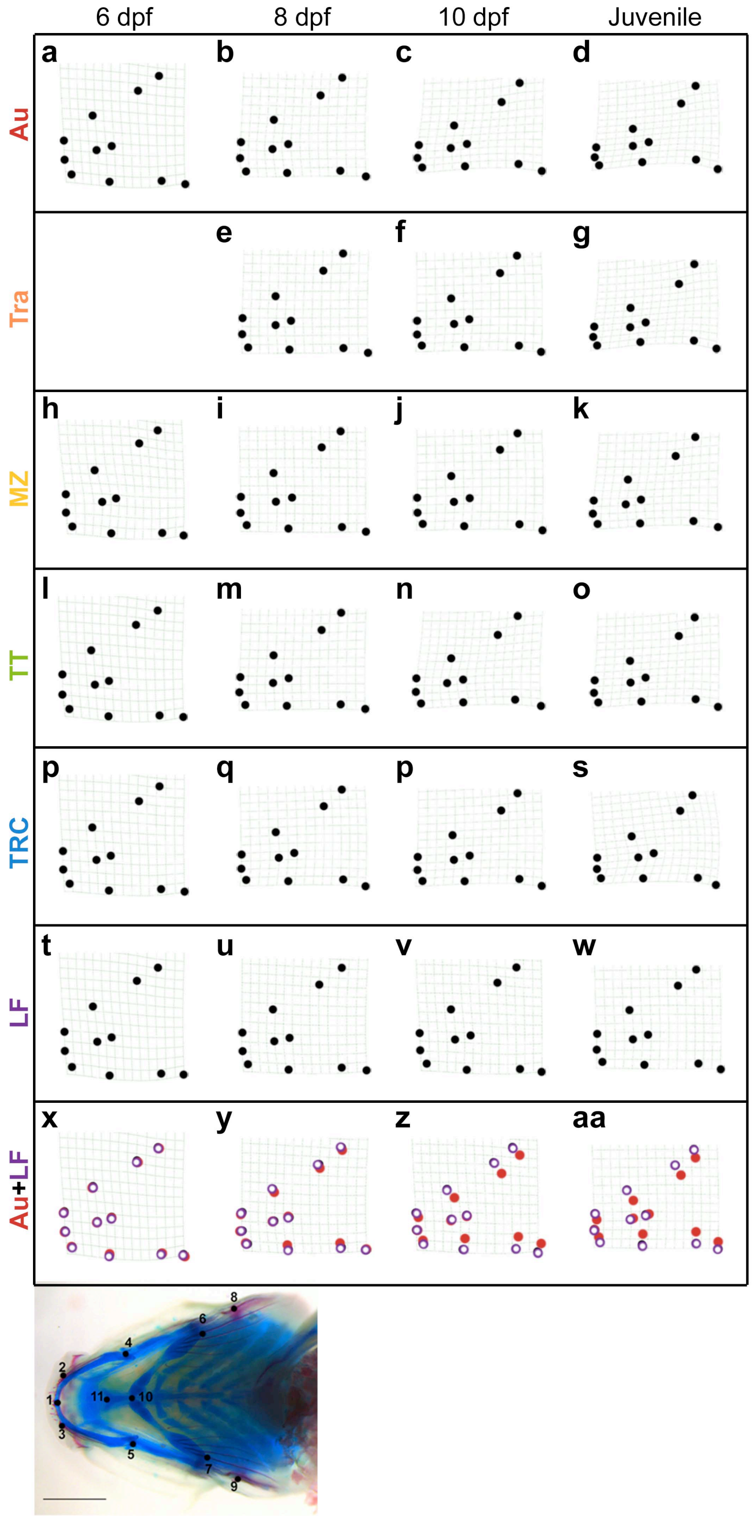

Figure S7. Species-specific ventral development. Deformation grids representing mean PC1 and mean PC2 score for each species at each developmental timepoint: **(a-d)** Au, **(e-g)** Tra, **(h-k)** MZ, **(l-o)** TT, **(p-s)** TRC, and **(t-w)** LF. **(x-aa)** Overlay of Au in red and LF in purple at each developmental stage.

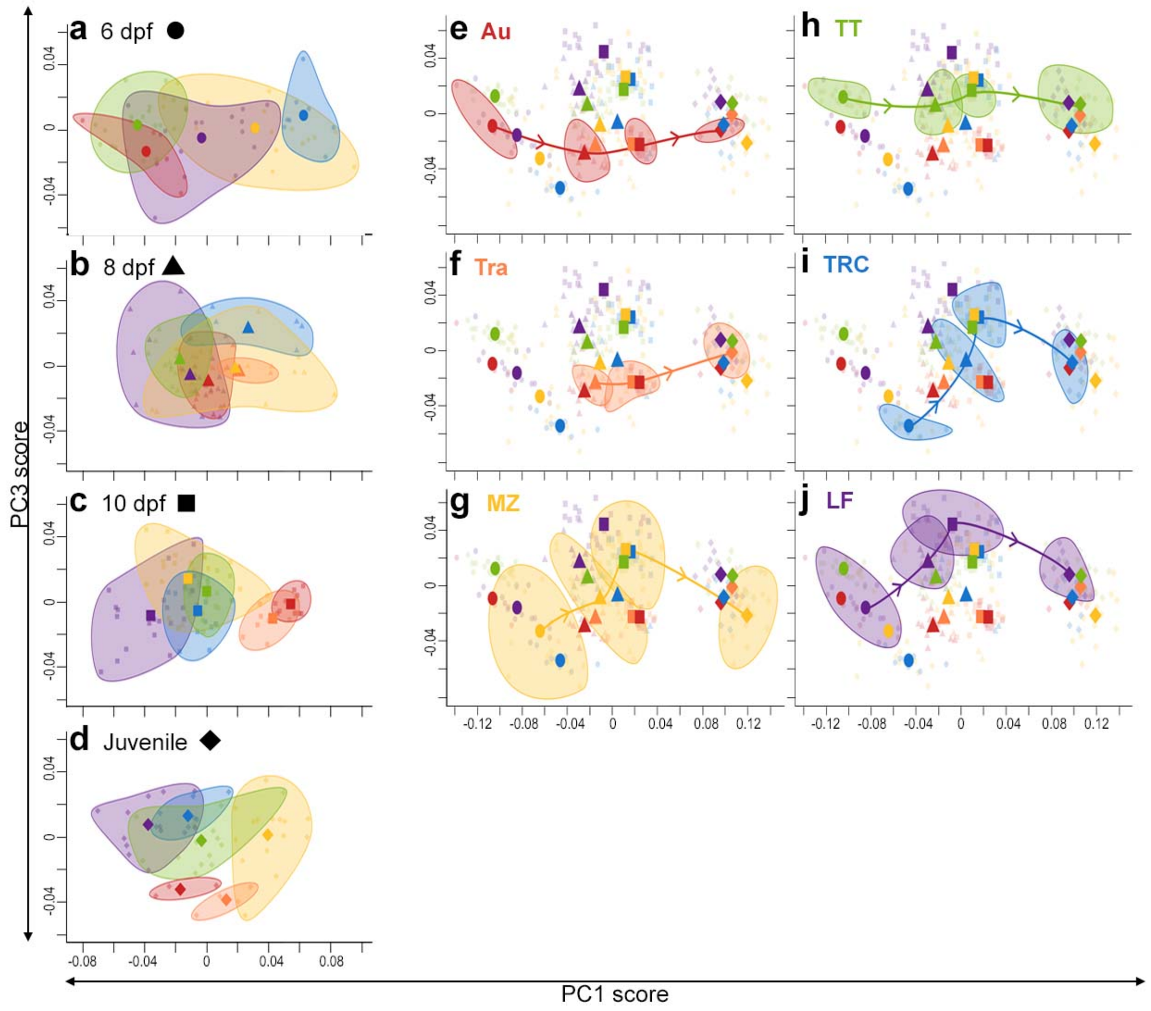

Figure S8. Lateral morphospace for PC1 and PC3. Differences in lateral shape are described by degree of facial outgrowth, particularly the preorbital region, and commensurate changes in craniofacial slope (PC1) and outgrowth in the posterior region of the neurocranium (PC3). Differences were analyzed both **(a-c)** within a single day and **(d-i)** over ontogeny. Colors, symbols, and ellipses are as described in Figure 3.

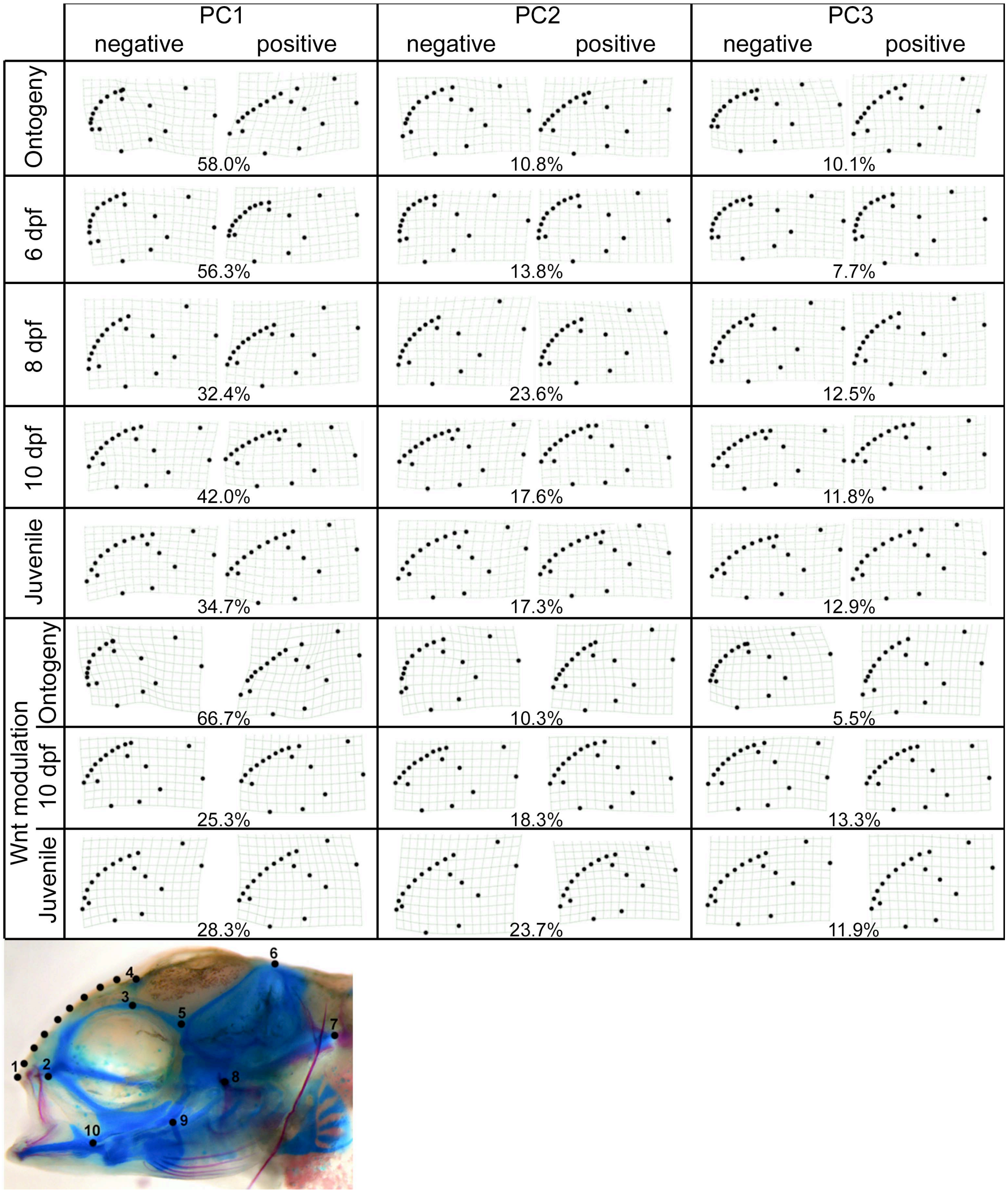

Figure S9. Deformation warps for lateral development. Visualization of lateral shape changes and percent of total shape variation described by principal components 1-3, analyzed both within a single day and through ontogeny.

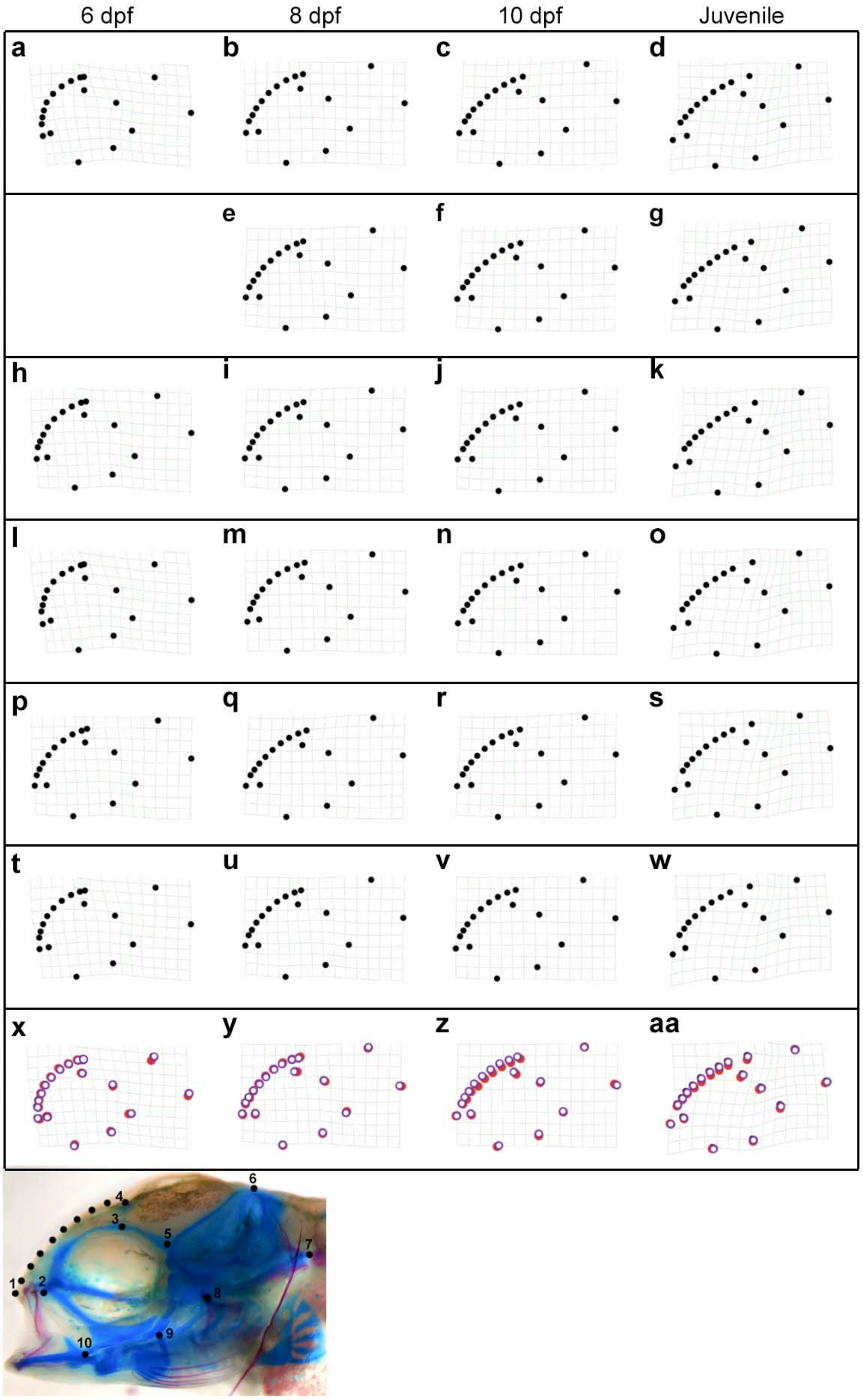

Figure S10. Species-specific lateral development. Deformation grids representing mean PC1 and mean PC2 score for each species at each developmental timepoint: **(a-d)** Au, **(e-g)** Tra, **(h-k)** MZ, **(l-o)** TT, **(p-s)** TRC, and **(t-w)** LF. **(x-aa)** Overlay of Au in red and LF in purple at each developmental stage.

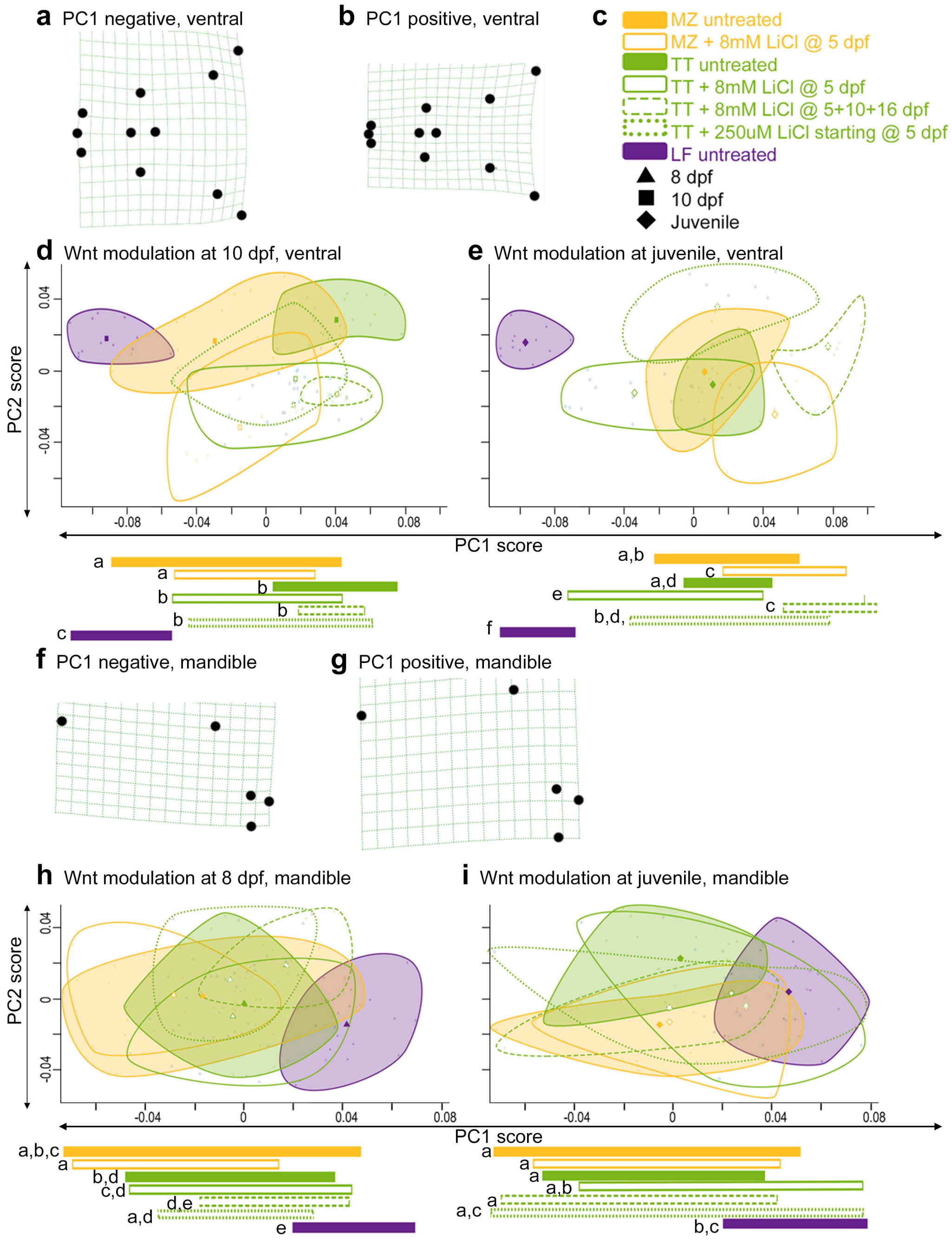

Figure S11. Wnt modulation does not alter ventral or mandible developmental trajectories. (a-b) Variation in ventral shape described by PC1 at juvenile stages following Wnt modulation. PC1 at 10 dpf describes similar variation (see Fig. S6). **(c)** Legend of colors and symbols used. Ventral morphospace at **(d)** 10 dpf and **(e)** juvenile stages. **(f-g)** Variation in mandible shape described by PC1 at juvenile stages following Wnt modulation. PC1 at 10 dpf describes similar variation (see Figs. S2 and S6). Mandible morphospace at **(h)** 10 dpf and **(j)** juvenile stages. Bars underneath plots indicate range of PC1 scores for each species/treatment. Statistical grouping based on ANOVA with Tukey's HSD using a cutoff of p=0.05.
